# Evaluating the impact of scoring parameters on the structure of intra-specific genetic variation using RawGeno, an R package for automating AFLP scoring

**DOI:** 10.1186/1471-2105-10-33

**Published:** 2009-01-26

**Authors:** Nils Arrigo, Jarek W Tuszynski, Dorothee Ehrich, Tommy Gerdes, Nadir Alvarez

**Affiliations:** 1 Laboratory of Evolutionary Botany, Institute of Biology, University of Neuchâtel, 11 rue Emile-Argand, CH-2000 Neuchâtel, Switzerland; 2 Science Applications International Corporation (SAIC), 1710 SAIC Drive Suite 3155 McLean, VA 22102, USA; 3 Department of Biology, University of Tromsø, N-9037 Tromsø, Norway; 4 Chromosome Laboratory, Department of Clinical Genetics, Rigshospitalet, Blegdamsvej 9, Copenhagen, Denmark; 5 Laboratory of Evolutionary Entomology, Institute of Biology, University of Neuchâtel, 11 rue Emile-Argand, CH-2000 Neuchâtel, Switzerland

## Abstract

**Background:**

Since the transfer and application of modern sequencing technologies to the analysis of amplified fragment-length polymorphisms (AFLP), evolutionary biologists have included an increasing number of samples and markers in their studies. Although justified in this context, the use of automated scoring procedures may result in technical biases that weaken the power and reliability of further analyses.

**Results:**

Using a new scoring algorithm, RawGeno, we show that scoring errors – in particular "bin oversplitting" (i.e. when variant sizes of the same AFLP marker are not considered as homologous) and "technical homoplasy" (i.e. when two AFLP markers that differ slightly in size are mistakenly considered as being homologous) – induce a loss of discriminatory power, decrease the robustness of results and, in extreme cases, introduce erroneous information in genetic structure analyses. In the present study, we evaluate several descriptive statistics that can be used to optimize the scoring of the AFLP analysis, and we describe a new statistic, the information content per bin (I_bin_) that represents a valuable estimator during the optimization process. This statistic can be computed at any stage of the AFLP analysis without requiring the inclusion of replicated samples. Finally, we show that downstream analyses are not equally sensitive to scoring errors. Indeed, although a reasonable amount of flexibility is allowed during the optimization of the scoring procedure without causing considerable changes in the detection of genetic structure patterns, notable discrepancies are observed when estimating genetic diversities from differently scored datasets.

**Conclusion:**

Our algorithm appears to perform as well as a commercial program in automating AFLP scoring, at least in the context of population genetics or phylogeographic studies. To our knowledge, RawGeno is the only freely available public-domain software for fully automated AFLP scoring, from electropherogram files to user-defined working binary matrices. RawGeno was implemented in an R CRAN package (with an user-friendly GUI) and can be found at .

## Background

For the past decade, studies on ecology and evolution have relied upon the assessment of genetic diversity in populations and species [1]. Genomic screening approaches for the measurement of diversity are more satisfactory than any other phenotype- or genotype-based techniques in the sense that they reveal a large number of markers [2]. Before the 1990's, restriction fragment length polymorphisms (RFLP [3]), random amplified polymorphic DNA (RAPD [4]) and simple sequence repeats (SSR or microsatellites [5]) were widely used to generate a relatively high number of markers. However, the implementation of amplified fragment length polymorphisms – AFLP [6], a relatively cheap, easy, fast and reliable method [7] – has exponentially increased the number of informative markers, resulting in large datasets. Most of those approaches retrieve genetic information through PCR-based techniques coupled with electrophoretic gels. As a result, markers are identified according to their absolute size that is measured as a function of mobility. The AFLP technique starts by digesting genomic DNA with two restriction enzymes (*EcoR *I and *Mse *I according to the original protocol [6]). This step is followed by the amplification of a subset of the restricted DNA fragments (requiring several intermediate steps, see [7]) through two successive PCR reactions (namely, the preselective and selective PCRs) and the separation of the amplicons by electrophoresis. Amplicons are fluorescently labelled and electrophoresis takes place in a genotyping machine, for instance using the GeneScan technology (i.e. amplicons migrate along a capillary during a span of time proportional to their size). As a result, the reaction conducted on a sample constitutes an "AFLP electropherogram" or "profile", in which each amplicon is recorded as a "peak" that is characterized by its mobility (converted to size and measured in base pairs, "bp") and intensity (measured as relative fluorescence units, "rfu"). The final step of the analysis aims to convert numerous AFLP profiles, that reflect the results of an AFLP reaction conducted on many samples, into a binary matrix where the presence/absence of each amplicon is recorded for each sample. While the GeneScan technology improves the accuracy of the genotyping process, its precision is not absolute and several factors (i.e. biases occurring during restriction, amplification or migration of the fragment in the capillary of the genotyping machine [8]) can affect the recorded size of an amplicon. Consequently, variability of recorded sizes complicates the analysis, as the same amplicon may have similar but non-equal sizes throughout the sampling. Due to these variations, checking and recording the presence/absence of a given amplicon, through the sampling, is generally done manually by the user. This phase is termed "scoring" and is achieved by the following procedures:

I. Defining amplicon size categories (i.e. called "bins") that ideally represent AFLP loci.

II. Recording the presence/absence of an amplicon within each bin and for each sample [9]. As a result, an AFLP locus will be coded as a binary state, where the presence of an amplicon is coded with 1 ("present" allele) while the absence of the amplicon is coded as 0 ("null" allele).

Programs such as Genographer V2.0 [10] (freely available at  [verified on December 30, 2008]) propose a graphical solution by displaying the GeneScan results on a "gel-like" interface and allowing the user to manually define the bins. Although this strategy allows direct control of the scoring, it requires experienced users and remains sensitive to human biases (e.g. see Bonin et al. [11]). Moreover, the procedure becomes problematic for large numbers of samples and adding new samples to the analysis requires a new scoring session. As a consequence, the final results may vary among runs, among users and across time, weakening the reproducibility and reliability of the analysis. These limitations justify the use of partially automated (e.g. Peakmatcher V6.1 [12], freely available at  [verified on December 30, 2008]) or fully automated scoring procedures (e.g. GeneMapper V3.7, Applied Biosystems, Foster City, CA), where the user does not directly score the dataset, but parameterizes a scoring algorithm. At least three main issues must be considered when scoring a dataset (either manually or using automated procedures).

I. Size homoplasy [13], which can arise in two ways: a) when two non-identical amplicons are considered to be homologous because they display identical mobility or b) when two amplicons are scored as absent at the same locus for differing reasons (e.g. amplicon size polymorphism or mutation in the restriction site).

II. Occurrence of bin definition errors resulting from a non-optimal scoring parameterization. This bias can lead to two contrasting errors: either a) "oversplitting", in which bins are too thin and may split variant locations of the same amplicon into smaller and erroneous sub-bins, or b) including an exaggerated range of amplicon sizes within the same bin, thus introducing an artificial similarity between unrelated samples. Although they differ in their causes, we assume that this second bias has comparable consequences for the dataset quality as those caused by size homoplasy. We will therefore term this bias "technical homoplasy" in order to distinguish it from size homoplasy.

III. Difficulties in detecting amplicons due to variable quality of AFLP reactions. Low quality runs can lead to the introduction of a noisy signal (i.e. "false-negatives" or "false-positives") within the dataset. This bias can be limited by using optimized and standardized laboratory AFLP protocols [8] and by running blank samples in order to determine the background noise associated with the genotyping machine.

In addition, recent studies propose the evaluation of the quality of both bins and alleles in order to increase the final dataset quality. Several of these procedures are applied once the scoring is complete [14]. However, it is also possible to proceed before the scoring phase, while analysing the AFLP profiles, for instance by tuning the peak detection parameters [15].

The scope of the present study covers methodology of automated AFLP scoring in the framework of population genetics and phylogeography. We propose a new automated solution for scoring AFLP electropherograms: RawGeno, a program implemented as a package in the widely used R CRAN freeware. We investigate the effects of sub-optimal settings on our algorithm, by focusing on two upstream processes of the AFLP electropherograms analysis: the bin definition and the recording of alleles. For this purpose, we tuned scoring parameters and produced five datasets differing in the average width of bins. This strategy, applied to the model-species *Cerastium uniflorum *(Caryophyllaceae), produced five datasets with increasing technical homoplasy. In parallel, we produced five analogous datasets with the commercial software GeneMapper. Finally, we scored the dataset manually by using the freeware Genographer. Using these eleven datasets, we first evaluate several descriptive statistics that can be used to optimize the scoring of the AFLP data. We then investigate the effects of the AFLP scoring settings, as well as the choice of the scoring method, on downstream analyses such as data mining statistics (ordination techniques), inter-individual and inter-population distance, Maximum Likelihood clustering (using PSMix [16], an algorithm aimed to investigate patterns of genetic structure) and population diversity indices.

## Methods

### Technical features of RawGeno

The analysis begins by detecting and calculating the size of peaks within the AFLP profiles. This preliminary analysis is conducted either with GeneScan V3.1.2 (ABI) or with the freeware PeakScanner V 1.0 (ABI,  [verified on December 30, 2008]) to produce an exhaustive list of detected amplicons generated by the AFLP reaction. This list records the size, fluorescence and sample origin of each amplicon which are used as the input data for RawGeno. It should be noted that our program can potentially be modified to handle results from other genotyping machines.

**Figure 1:**
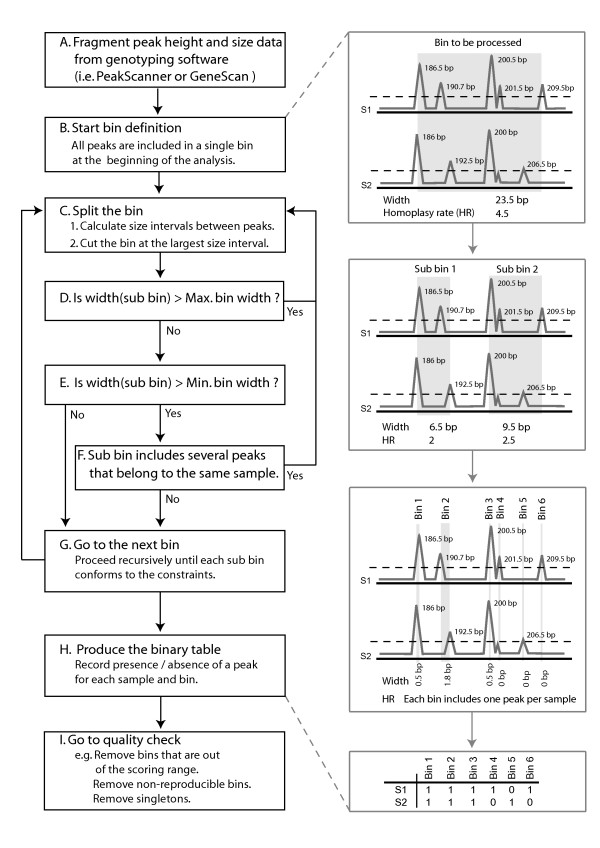
**Flowchart illustrating the bin definition algorithm of RawGeno**. On the right side: an example showing two samples (S1 and S2) where a total of nine AFLP peaks were preliminarily detected and sized. The bin width (i.e. the difference in size between the longest and the shortest amplicons included in the considered bin) and the technical homoplasy (i.e. the mean number of peaks belonging to the same sample that are included in a same bin) are indicated for each bin.

The bin definition algorithm [17] is the core of our library (see Figure 1. for a thorough explanation). In brief, it begins by defining the locations and limits of the bins. The bins are identified on the basis of the range of amplicon sizes from all samples, regardless of their fluorescence intensity (amplicons are preliminarily filtered according to their intensity during the detection and sizing analysis; e.g. 50 rfu as in our present study). This strategy aims to minimize the bin widths while maximizing the intervals between bins. Amplicons are sorted according to their sizes and size intervals are computed between each consecutive amplicon. Transitions between successive bins are identified by considering the inter-bin size intervals to be larger than the intra-bin ones. Two additional rules are involved in bin definition. First, bins must fit within a user-specified width range (i.e. the "maximum bin width" is parameterized). Second, a bin can include only one amplicon per sample. The application of this second rule is modulated by the "minimum bin width" parameter, in order to allow the occurrence of technical homoplasy. As a result, "thin" bins are accepted only if they effectively co-occur in at least one sample. Similarly, the definition of "wide" bins that would include more than one amplicon per sample within a single bin is prevented as often as possible but remains possible by manipulating the "minimum bin width" parameter. Once bins are defined, the algorithm notes the presence or absence of an amplicon in each bin for each sample and builds a binary matrix. Finally, amplicon features (i.e. fluorescence and size) are used to improve the binary matrix quality (see Additional file 1).

### Empirical data and experimental design

In order to investigate the effects of the scoring procedure on genetic analyses, a dataset from an extensive study on intra- and inter-specific plant biodiversity (IntraBiodiv Consortium [18]) was chosen as a model. This dataset includes samples covering the whole geographic range of *Cerastium uniflorum *(Caryophyllaceae), a perennial, diploid (2n = 36) plant distributed throughout the European Alps in subnival habitats. A total of 209 individuals (including 40 individuals that were replicated in the DNA extraction step) from 46 populations (four individuals per population on average) were analysed with three selective AFLP primer pairs. Details on the sampling scheme, the primer pairs used and the scoring methods are provided in Gugerli et al. [18] and Bonin et al. [19]. Raw data were obtained after running AFLP reactions on an ABI 3100 sequencing machine (ABI) and analysing electropherograms using GeneScan V3.1.2 (ABI). The program was set up with default detection parameters; only peaks ranging between 50 and 500 bp with a minimum fluorescence intensity of 50 rfu (i.e. the minimum reportable peak height according to Gilder et al. [20]) were included in the analysis. In the context of the Intrabiodiv Consortium, the scoring was performed manually using Genographer V1.6 [10], through a classical user-defined protocol [18], in which non-reproducible bins were discarded before being recorded (as proposed in Bonin et al. [11]). As a consequence, this manually scored dataset did not include information regarding rates of reproducibility of alleles and individuals. The resulting scored matrix (referred to as "manual" below) was coded as binary states with lines and columns recording presence or absence of amplicons in samples and bins respectively. Finally, ten datasets were produced by tuning the scoring parameters of RawGeno and GeneMapper. We tuned the "minimum bin width" parameter of RawGeno and the "bin width" parameter of GeneMapper in order to force the algorithms to assign amplicons displaying close but differing sizes to the same bin. As a consequence, the technical homoplasy of the datasets increased proportionally while increasing the bin width.

### Automated scoring procedures

The parameters of RawGeno were determined as follows (refer to Figure 1. for a scheme of the scoring algorithm and its parameters): the "maximum bin width" was left unconstrained and set as being the maximum observed amplicon size in the dataset (default value from the original algorithm). As a consequence, RawGeno avoided systematically the oversplitting bias (see above). In contrast, the "minimum bin width" was constrained during the analysis and set to 0.2 bp, 1 bp, 2 bp, 5 bp and 10 bp. This strategy produced datasets with an increasing amount of technical homoplasy since RawGeno was forced to use wider bins. It bears repeating that this setting strategy was used to intentionally increase the technical homoplasy in the produced datasets and not to produce optimally scored datasets (refer to the Additional file 1 for recommendations to conduct a proper scoring with RawGeno). The parameters in GeneMapper were determined using the standard detection settings and a polynomial degree of three was used for peak recognition in the electropherograms. The scoring step was achieved by tuning the "bin width" parameter with values ranging from 0.2 bp to 10 bp (identically to RawGeno settings). The precise effect of the "bin width" parameter on the GeneMapper scoring algorithm could however not be predicted *a priori *and was deduced from the results we obtained. Replicated samples (i.e. ~20% of the sampling) were included in all ten datasets, which allowed the calculation of error rates (see below) and subsequently, the removal of non-reproducible bins (as done during the manual scoring) in the final dataset. Monomorphic bins and singletons were also removed. The whole set of downstream analyses (see below) were carried out on these cleaned datasets. The ten resulting matrices were coded as binary states in the same way as for the manual dataset (see above). For RawGeno, datasets were labelled as follows: RG_0.2, RG_1, RG_2, RG_5 and RG_10. GeneMapper datasets were labelled as follows: GM_0.2, GM_1, GM_2, GM_5 and GM_10.

### Descriptive statistics

Several indices were computed. First, the final number of bins (nbin) was recorded for each dataset. Second, the mean homoplasy rate was computed within the RawGeno datasets. The homoplasy rate (HR) was defined as the number of amplicons belonging to the same individual that are assigned within the same bin. This statistic was calculated for each sample/bin and averaged for the whole dataset. The frequency of the "present" allele was computed for each bin and frequencies were plotted against the bin sizes (in bp). Finally, the level of correlation was calculated between each of the ten automatically scored datasets and the manually scored one (i.e. performing Pearson's correlations; hereafter referred to as "R2 Manual"). This was achieved by: I. Calculating Jaccard similarity indices [21] between samples, within each dataset. This calculation is defined as being asymmetric as it only accounts for presences in individual genotypes while absences are not considered. II. Calculating Pearson's correlation between the resulting similarity matrices, using the similarity matrix obtained with the manually scored dataset as reference.

### Ordination techniques

The datasets produced from the automated analysis were compared to the manually scored one by using a partial constrained correspondence analysis [21]. We used the "vegan" package [22] implemented in the R CRAN environment and applied the "cca" function (using a "scaling 1" procedure, in order to optimally represent the samples coordinates). This analysis was used to produce residuals containing information that is specific to the automatically scored dataset, according to the following model: V_a _- V_m _= R_a_, where V_a _is the variance of the automatically scored dataset, V_m _is the variance of the manually scored dataset and R_a _is referenced to as the residuals that are specific to the automatically scored dataset. We further measured the ability of these residuals to discriminate populations, in order to assess whether the automatically scored datasets effectively contained more information than the manually scored one. This calculation was achieved by applying a Mantel test between the residuals matrix and a contrast matrix comprising the population origin of each sample. This test required the computation of Euclidean distance on the contrast and residual matrices, for which we used the "mantel" function of the "vegan" R CRAN package (1000 permutations).

### Error rates and optimality criterion

For each dataset, two estimators of the error rate were computed by considering the information comprised in the replicated samples.

I. The mismatch error rate [11] defined as E_b _= M_repl_/nbin, where M_repl _is the total number of mismatches between a sample and its replicate and nbin is the total number of bin. This statistic was computed for each sample-replicate pair.

II. The Bayesian error rates ε1.0 and ε0.1 that represent, respectively, the probability of mis-scoring the presence or the absence of AFLP fragments. We used MasterBayes (an R CRAN package [23]) and the AFLPScore R CRAN script collection [14] to compute 1000 estimates of these statistics.

These two estimators required the inclusion of replicated samples and did not allow addressing the quality of datasets where non reproducible bins had been removed (e.g. in the manually scored dataset). As a consequence, we propose a new "optimality criterion" based on the information content per bin (I_bin_). This statistic was calculated for each sample of the dataset and was defined as I_bin _= M_sampling_/nbin where M_sampling _is the average number of mismatches between the considered sample and the other samples of the dataset and nbin is the total number of bins in the dataset. This criterion can be computed at any stage of the scoring process and does not require the inclusion of replicated samples. Here, we applied it after the removal of non-reproducible bins.

### Biogeographic structure

We investigated the spatial genetic structure in our datasets at two levels of complexity: first between individuals by computing the Jaccard similarity index [21], second between populations by using an estimator of the FST and by performing Maximum Likelihood clustering (assuming Hardy-Weinberg equilibrium). FSTs were computed with the program AFLP-Surv [24] (allelic frequencies were estimated with a Bayesian method using a non-uniform prior distribution and assuming Hardy-Weinberg equilibrium). Jaccard similarity indices (see above) and FST values obtained with the various datasets were compared using scatterplots and linear regressions. Maximum Likelihood clustering was performed using PSMix [16], a package implemented under the R CRAN environment. It uses Maximum Likelihood methods in order to assign individuals into a predefined number of groups with an associated probability. The algorithm assumes Hardy-Weinberg equilibrium within groups and linkage equilibrium between loci. The datasets were coded as follows: each individual genotype was duplicated, in order to simulate a diploid co-dominant dataset. The absences (0) of the original genotype were coded as absences in the duplicated genotype (i.e., "0" is coded as "0-0") whereas presences (1) occurring in the original genotype were coded as missing data in the duplicated genome (i.e., "1" is coded as "1-?"). This coding scheme is adapted from Bonin et al. [19]. The default settings of PSMix were applied (except for itMax [i.e. the maximum number of iterations] that was set to 100000). The number of investigated groups (K) ranged from two to nine, with ten replicated runs per K. For each K value, only the run showing the highest likelihood value was selected for further analysis. The resulting assignment probabilities were compared using pie-charts mapped on geographical maps.

### Diversity indices

Three indices, revealing the level of diversity in each population, were computed: I. the estimated Heterozygosity (Hj), by using AFLP-Surv [24] (we used the same parameters as above). II. The percentage of polymorphic loci (PLP), using AFLPdat, an R CRAN script collection [25]. III. The presence/absence rarity index (i.e. "rarity 2" according to the IntraBioDiv Consortium's [18] explanations at  [verified on December 30, 2008]). The values estimated with the various datasets were compared using scatterplots and linear regressions.

## Results and discussion

### Applying the scoring

**Figure 2:**
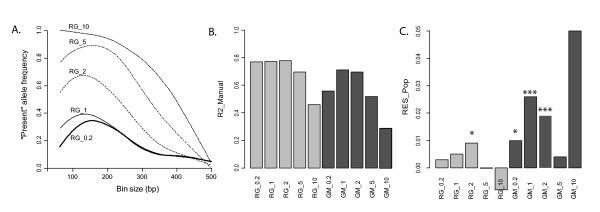
**General statistics**. In light grey, RawGeno datasets labelled with "RG" as prefix; in dark grey, GeneMapper datasets labelled with "GM" as prefix. A. Comparison of the frequency of the "present" allele (i.e. the occurrence of an amplicon within a bin) according to the bin size, in datasets scored with an increasing bin width using RawGeno. B. Pearson's correlation calculated between the automatically scored dataset and the manually scored one (R2_Manual). C. Partial constrained analysis results. This data mining technique allows the removal of the variation explained by the manually scored dataset in the automatically scored one. As a result, the residuals contain information that is specific to the automatically scored dataset. RES_Pop is a measure of the discrimination power of these residuals in identifying populations. We computed and tested Pearson's correlations between the residuals and a contrast matrix indicating the population origin of samples (Mantel test, 1000 permutations). Significance levels: * (p < 0.05), ** (p < 0.01) and *** (p < 0.001).

The manual scoring required approximately 100 hours of "human work" and produced 102 reproducible bins. Automated scoring procedures, in contrast, produced the scorings in about 1 hour (depending on the kind of dataset and the computer power). Moreover, the automatic scoring procedures preserved a large number of bins as we obtained 502, 456, 316, 177 and 116 bins for the RawGeno datasets (RG_0.2 to RG_10) and 4126, 1338, 742, 340 and 183 bins for the GeneMapper datasets (GM_0.2 to GM_10). As expected, the use of larger bin widths decreased the number of bins and introduced technical homoplasy. Interestingly, technical homoplasy occurred more rapidly, with the increase of the bin width, in small fragment sizes (i.e. sizes < 200 bp, Figure 2A) than in larger fragments. This result is explained as follows. Amplicon sizes are generally asymmetrically distributed because short amplicons are often over-represented compared to larger ones (and especially in genomes with a low GC content when *EcoR *I and *Mse *I are used as restriction enzymes) [13,26]. As a consequence, these small amplicons were shown to be especially prone to reflect size homoplasy [13] and in addition, our results showed that they were also likely to accumulate more technical homoplasy than the rest of the AFLP profile.

### Using manual scoring as a reference point

We first investigated the quality of datasets by referring to the manually scored dataset. Technical homoplasy, generated by forcing the scoring algorithm to create large bins (i.e. RG_5, RG_10, GM_2, GM_5 and GM_10), clearly impacted the dataset quality, as shown by a decreasing correlation to the manual dataset with increasing bin width (see Figure 2B). This result matched our expectations and outlined the effects of technical homoplasy. Interestingly, we observed an optimal bin width effect in the GeneMapper datasets (Figure 2B), where datasets scored with small bin widths showed less correlation to the manually scored one than the datasets scored with a greater bin width (i.e. GM_0.2 and GM_1 showed correlations of 0.558 and 0.713, respectively, with the manually scored dataset). Conversely, in RawGeno this phenomenon was avoided since correlation values remained relatively constant between datasets that were scored with small to medium bin widths (i.e. RG_0.2, RG_1 and RG_2 showed correlations of 0.771, 0.774 and 0.779, respectively, with the manually scored dataset). Divergences between RawGeno and GeneMapper can probably be explained by differences in algorithm settings. While RawGeno allowed the user to set both the "minimum" and the "maximum bin width", we suspect that the "bin width" parameter of GeneMapper might have effects similar to those of the "maximum bin width" parameter of RawGeno. Decreasing the "maximum bin width" parameter can exaggerate the splitting of bins and, as a result, appropriately sized bins might be split into smaller and erroneous sub-bins. This bias is expected to produce datasets with a high number of low-quality bins ("oversplitting of bins"). Such a hypothesis is in accordance with the very high number of bins produced in GM_0.2 and GM_1 for instance. Holland et al. [15] showed that, by using GeneMapper, choosing a bin width below 0.4 bp was misleading since it resulted in oversplitting. We could however not obtain an absolute confirmation of this hypothesis since GeneMapper algorithms are strongly black-boxed.

Divergences related to varying bin width parameters were also detected by the partial constrained correspondence analysis (Figure 2C). This procedure inspected the residuals of the automatically scored datasets (after having removed the variation explained by the manually scored dataset). It appeared that the residuals contained relevant information in several datasets (e.g. the residuals of RG_2, GM_0.2, GM_1 and GM_2 significantly discriminated populations). This was particularly true for GeneMapper datasets where the scoring algorithm outlined several additional biogeographic patterns (see Additional file 2). These patterns, however, were seldom interpretable since they segregated single populations from the rest of the samples and were identified neither by the manual scoring nor by RawGeno (see below). We could however consider that the very high number of bins associated with these GM datasets (GM_0.2, GM_1 and GM_2) might have included such additional private alleles. Finally, both oversplitting and technical homoplasy decreased the information content of residuals. This result was reinforced by an optimum bin width effect where only RG_2, from the RawGeno datasets and GM_1 and GM_2 from the GeneMapper datasets showed the highest residual information content, while technically biased datasets presented lower values.

### Error rates and optimality criteria

**Figure 3:**
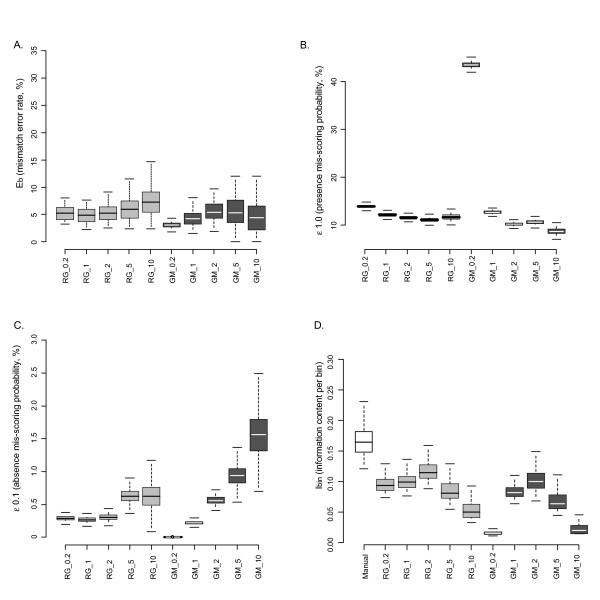
**Error rates and information content**. In light grey, RawGeno datasets labelled with "RG" as prefix; in dark grey, GeneMapper datasets, labelled with "GM" as prefix. Only the quartiles are displayed on the box-plots. A. Mismatch error rate (E_b_). B. and C. Bayesian error rates representing the probability (%) of mis-scoring allele presence (ε1.0) or absence (ε0.1). D.Information content per bin (I_bin_), calculated as the mean inter-individual distance divided by the number of bins in the dataset. This statistic could also be computed for the manually scored dataset since it does not require replicated samples.

The previous statistics used the manually scored dataset as a reference. This strategy, however, might be misleading for several reasons. First, this reference point is not available when analysing a new dataset and second, manual scoring may introduce subjective biases (for instance resulting from different filtering strategies) in the dataset. It is therefore clear that the evaluation of dataset quality requires a much more absolute criterion, such as error rates. However, the properties of the different estimators must be evaluated taking into account technical homoplasy and bin oversplitting. For instance, the mismatch error rate (E_b_), defined by the number of mismatches between a sample and its replicate, divided by the number of bins (E_b _= M_repl_/nbin), failed to unambiguously detect the oversplitting, while technical homoplasy was slightly detected (Figure 3A). On one hand, oversplitting could not be detected by an increase of the mismatch error rate since it dramatically increased the number of bins. Technical homoplasy, on the other hand, was hardly detected because it artificially decreased the number of mismatches between a sample and its replicate, leading to an underestimation of E_b_. Similar results were described by Holland et al. [15] where the E_b _rate (i.e. referred to as the Euclidean Error rate by the authors) was shown to be unable to discriminate datasets scored with varying bin sizes. As a result, we advise that the mismatch error rate should not be used to optimize the bin definition and the scoring of AFLPs. This criterion provided, however, valuable information when the number of bins remained similar among datasets. This situation was encountered, for instance, during further quality checking steps, such as fluorescence or bin reproducibility filtering. Interestingly, the two Bayesian error rates (Figure 3B and 3C) detected both the oversplitting (with ε1.0) and the technical homoplasy (with ε0.1). These indices thus provided a valuable solution for optimizing the whole range of scoring parameters, including downstream filtering procedures as shown by Whitlock et al. [14]. These statistics, however, required a large computational time and a quicker alternative may be desirable. We therefore propose the use of a new statistic, i.e. the information content per bin (Figure 3D), as a quality criterion to optimize the first steps of AFLP scoring (see above for explanations regarding this new criterion). In our study, this statistic detected both the oversplitting and technical homoplasy. Oversplitting increased the number of bins faster than the number of mismatches. In contrast, the technical homoplasy decreased the accuracy of the dataset at a faster rate than the number of bins decreased. Maximizing the I_bin _represents an interesting trade-off between the accuracy of the dataset and the number of bins that are used to record the AFLP information. We propose applying this criterion to optimize the bin definition, while the other quality criteria (e.g. Whitlock et al. [14]) can be used during downstream scoring steps. According to the I_bin _criterion, we assumed that RG_2 and GM_2 datasets had a reasonably good quality. This result was confirmed for RG_2 by both the correlation to the manually scored dataset and the partial constrained correspondence analysis (see Figure 2), while according to these same statistics, GM_1 might perform better than GM_2. It bears repeating, however, that datasets scored with bin widths up to 2 bp probably included some level of technical homoplasy. The presence of stutter-bands (i.e. PCR artefacts) might contribute to this. The effect of this phenomenon on scoring could either produce rare alleles that led to the definition of non-informative bins or cause erroneous results when the range of the stutter bands extended into another bin (e.g. oversplitting bins or mis-scoring of absences or presences). Moreover, stutter-bands generally had a decreased fluorescence and their various peaks might not be reproducible. Therefore, scoring parameters that considered a cluster of stutter-bands as a single allele probably handled the situation in a more appropriate way.

### Analysis sensitivity

**Figure 4:**
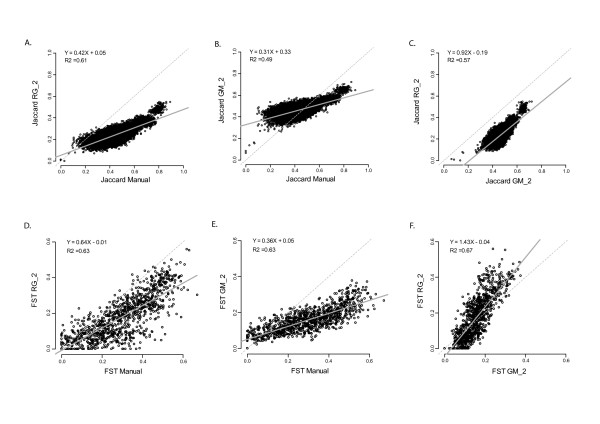
**Comparison of genetic structure estimators**. The statistics evaluate the consistency of two structure analyses, computed with the manual dataset and two automatically scored datasets assumed to be accurate according to the I_bin _criterion (i.e. "RG_2" and "GM_2"). The estimations obtained with the three datasets are compared with pairwise scatterplots (RG versus Manual, GM versus Manual and RG versus GM) and linear regressions (displayed as a solid line). The R^2^, the slope and the intercept of the regression are indicated. A. to C. Inter-individual similarities (Jaccard similarity index). D. to F. Inter-population distances (FST).

Biogeographic structure analyses were moderately affected by the scoring system (Figure 4). For instance, both inter-individual (Jaccard similarity index) and inter-population (FST) distances provided comparable results from one scoring method to the other (although RG datasets matched the manually scored ones better than did GM datasets). Additionally, these measures were also moderately affected by the scoring parameters in cases when technical homoplasy and oversplitting were avoided (for instance, convergent results were obtained with the manual scoring, RG_0.2, RG_1 and RG_2 or GM_1 and GM_2 respectively [data not shown]).

**Figure 5:**
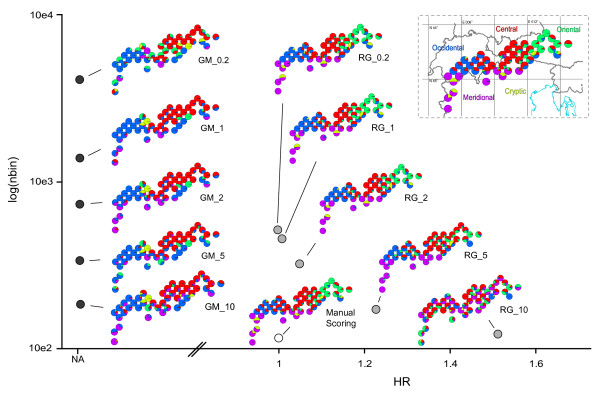
**Effect of the scoring parameters on the PSMix clustering analysis**. Scatterplot of the number of bins (nbin, log-scaled y-axis), according to the mean homoplasy rate (HR, x-axis). The mean homoplasy rate is defined as the average number of peaks belonging to the same individual that are affiliated within the same bin. The eleven datasets are displayed as dots (in dark grey, GeneMapper datasets labelled with "GM" as prefix; in light grey, RawGeno datasets labelled with "RG" as prefix and in white, manual dataset) and as maps on the scatterplot. The maps represent *C. uniflorum *populations with pie-charts representing assignment probabilities obtained with PSMix (K = 5 groups). On the upper-right corner: geographical location of the sampling and names of the identified phylogenetic groups (violet – Meridional cluster, blue – Occidental cluster, red – Central cluster, green – Oriental cluster and yellow – Cryptic cluster). The mean homoplasy rate could not be calculated for the GeneMapper datasets.

The PSMix clustering analyses provided similar patterns (Figure 5). The spatial genetic structure of *C. uniflorum *seemed to be composed of five main phylogeographic regions (the second-derivative of the Maximum Likelihood values showed a small decrease when investigating more than five groups [data not shown]; this result was also accompanied by a decrease in the clustering accuracy). These regions were identified by the clustering analysis in the following order (see Figure 5 and Additional file 2): Meridional (K = 2), Oriental (K = 3), Cryptic (K = 4), and finally Occidental and Central regions (K = 5). Interestingly, the automatically scored datasets showed much higher assignment probabilities to clusters than did the manual dataset (see Additional file 2). RawGeno and manual scoring provided congruent genetic structures while GeneMapper identified specific patterns that were difficult to interpret (as mentioned above). These patterns also disrupted the clustering analyses and similar values of K may not provide converging results when comparing the three scoring methods. The main phylogeographic structures were observed when inspecting the entire set of clustering runs (Additional file 2, with K ranging from two to nine), although results were slightly influenced by the scoring algorithm. The scoring parameters were also important. The consequences of technical homoplasy ranged from a loss of discrimination power (Figure 5 and Additional file 2) for the datasets with moderate homoplasy (e.g. RG_5), to erroneous results for the most biased ones (RG_10 which was largely non-informative). In this situation, coherent results could be observed only up to four groups (i.e. K = 4, Additional file 2) where the most evident phylogeographic regions were detected (Meridional and Occidental clusters) while detailed groupings (such as the Cryptic, Oriental and Central clusters) were missing or identified at higher values of K (see Additional file 2). Interestingly, and despite the relatively high number of bins (e.g. 177 bins for RG_5 vs. 102 for the manually scored dataset), accuracy of clustering was not observed beyond five groups in datasets displaying moderate homoplasy, indicating that the additional bins of these datasets probably contained noisy information (Additional file 2). This result also agreed with the I_bin _values that showed a decrease in the information content of these datasets.

**Figure 6:**
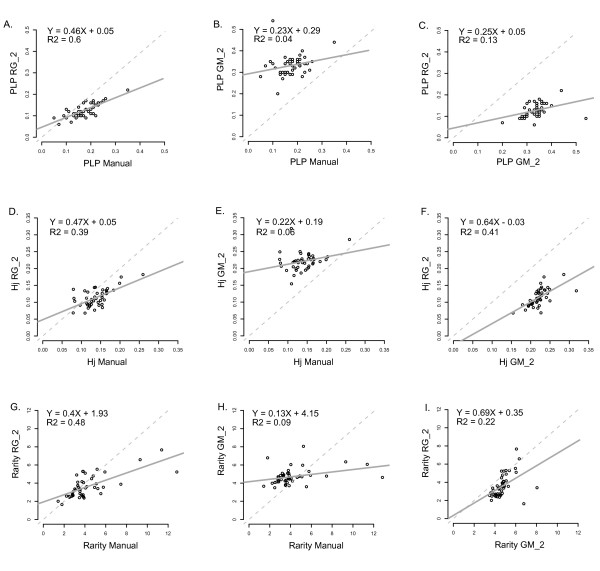
**Genetic diversity statistics**. The statistics evaluate the consistency of three diversity estimators calculated with the manual dataset and two automatically scored datasets assumed to be accurate according to the I_bin _criterion (i.e. "RG_2" and "GM_2"). The estimations obtained with the three datasets are compared with scatterplots (RG versus Manual, GM versus Manual and RG versus GM) and linear regressions (displayed as a solid line). The R^2^, the slope and the intercept of the regression are indicated. A. to C. Percentage of polymorphic loci (PLP). D. to F. Estimated Heterozygosity (Hj). G. to I. Rarity index (Rarity).

In contrast to genetic structure patterns, population diversity indices were extremely sensitive to the scoring features since the estimated Heterozygosity, the percentage of polymorphic loci and the rarity index showed important discrepancies among the datasets (Figure 6 and Additional file 3). Interestingly, the percentage of polymorphic loci and the estimated Heterozygosity were the most robust measures that we tested (Figure 6A to 6F). Detection of rare loci was more successful when the datasets were finely scored, but this statistic was particularly sensitive to the technical homoplasy (Figure 6G to 6H and Additional file 3C). We explain these results as follows. First, the sampling scheme of our model species might not be robust enough (with an average of four individuals per population) and in this context, estimates of genetic diversity might be strongly influenced by technical biases. Second, scoring biases such as the oversplitting of bins or the technical homoplasy affected the definition of bins and the recording of presence/absence of alleles. As a consequence, statistics that relied directly on the polymorphic status of presence/absence of alleles, (such as diversity measures at the within population level) were likely to be particularly sensitive to the dataset noise. As a result, scoring biases may have reinforced the problems caused by size homoplasy in estimating the genetic diversity [26]. This point raised the question of how to obtain reliable diversity estimates by using AFLP datasets and the use of other indices, such as the proportion of different genotypes in a population (not tested here because of the small sample sizes), might provide more robust results. In contrast, synthetic statistics such as distance-based methods or clustering analyses probably buffered the technical noise with the rich signal of AFLP and finally provided coherent results. Similar observations were made in other studies [15,19,26] where distance-based analyses proved to be robust regardless of the choice of the scoring algorithm and its settings. We also report convergent observations for gene flow detection (N. Arrigo and S. Lappe, unpublished data) where F1 hybrids and introgressed individuals were detected accurately, regardless of moderate levels of technical homoplasy. Finally, several steps that might help to eliminate unsatisfactory bins or peaks (such as filtering bins according to their average fluorescence [14]) were not applied in the present study. Better results may be obtained by optimizing this specific aspect of the analysis. Such filtering methods, as well as new tuning strategies, will be progressively implemented in RawGeno. In any case, however, we stress that users must carefully choose the parameters of the scoring algorithms to reflect the aim of the analysis.

## Conclusion

Scoring a dataset manually is a time consuming process that is complicated when investigating a large number of samples. Using an automated system can significantly increase the reproducibility of the dataset as all the electropherograms are scored uniformly and as genotyping errors are limited to technical factors (e.g. PCR or migration variations). Our study showed that, with high quality AFLP and GeneScan raw data, automated procedures can be particularly efficient in producing ready-to-use datasets, at least in the context of population genetic or phylogeographic studies (i.e., RawGeno was not tested in a genomics framework [e.g., for gene mapping] and further investigations are needed before validating its extension to this field). However, the automated scoring of AFLPs is a multiple-step process and a trade-off based on several quality criteria may be desirable since it might provide more relevant information than a single statistic. Optimizing the parameters used in the scoring algorithm therefore represents one of the most important steps of the whole analysis. Using RawGeno, we were able to evaluate the impact of technical homoplasy and bin oversplitting on optimality criteria and genetic structure patterns by intentionally biasing our starting datasets. Interestingly, our results demonstrated that a high number of redundant and informative bins might overcome technical homoplasy due to scoring errors, at least when investigating biogeographic structures. While allowing for some plasticity during the optimization of the scoring procedure, this result also reinforces the use of the AFLP methodology for its ability to produce highly informative datasets. By contrast, the estimation of genetic diversity may be considered with caution since scoring biases are likely to reinforce problems caused by size homoplasy.

Finally, RawGeno provided results at least as accurate as those obtained by scoring the dataset manually (even when considering bin widths as wide as 2 bp, representing an error range much higher than the technical error rate of the genotyping machines) or by using a commercial software such as GeneMapper. To our knowledge, RawGeno is the only freely available program proposing a fully automated scoring solution, from electropherogram files to user-defined working binary matrices. Benefiting from the open source R platform, RawGeno can be potentially enhanced and used by any user.

## Authors' contributions

NA1 carried out the main design of the study, organized the package, programmed and debugged the R code, and drafted the manuscript. JWT, DE and TG participated in the design of the study and programmed parts of the code. NA2 participated in the main design and coordination of the study and helped to draft the manuscript. All authors read and approved the final manuscript.

## Supplementary Material

Additional file 1 **Detailed technical features of RawGeno.** This document provides a description of scoring and bin filtering solutions proposed by RawGeno. In addition, it includes recommendations to achieve a proper scoring. Click here for file

Additional file 2 **Spatial genetic structures for all datasets.** The graphics are displayed on an array where the lines represent the different datasets (Manual – manual scoring, RG – RawGeno datasets and GM – GeneMapper datasets) and the columns display the different analyses. The three first columns contain the scatterplots of three genetic diversity indices (Rarity – rarity index, Hj – estimated Heterozygosity and PLP – percentage of polymorphic loci). The values obtained by using the automatically scored datasets (displayed on the y-axis) are compared to those obtained with the manually scored dataset (displayed on the x-axis). The red line represents a linear regression between values obtained by both datasets (the Pearson's correlation indices of these regressions are displayed in the Figure 6). The next columns contain individual clustering results (with the number of *a priori *groups ranging between K = 2 and K = 9). Click here for file

Additional file 3 ****Effect of the scoring parameters on diversity estimators.**** Scatterplot of the number of bins (y-axis, log-scaled), according to the mean homoplasy rate (x-axis). The mean homoplasy rate (HR) is defined as the average number of peaks belonging to the same individual that are affiliated within the same bin. The eleven datasets are displayed as dots (in dark grey, GeneMapper datasets labelled with "GM" as prefix; in light grey, RawGeno datasets labelled with "RG" as prefix and in white, manual dataset) and as maps on the scatterplot. The maps represent *C. uniflorum *populations with circles. The radius of circles is a function of the measured diversity. A. Percentage of polymorphic loci (PLP). B. Estimated Heterozygosity (Hj). C. Rarity index (Rarity). Click here for file
