# Supplementary material for: Evaluating the impact of scoring parameters on the structure of intra-specific genetic variation using RawGeno, an R package for automating AFLP scoring
Source: BMC Bioinformatics. 2009 Jan 26;10:33. doi: 10.1186/1471-2105-10-33 (PMC2656475; doi:10.1186/1471-2105-10-33)
Supplement: Additional file 1 — Detailed technical features of RawGeno. This document provides a description of scoring and bin filtering solutions proposed by RawGeno. In addition, it includes recommendations to achieve a proper scoring. [file 1471-2105-10-33-S1.doc]

**Additional file 1: Detailed technical features of RawGeno**

**A toolbox for analysing AFLP**

RawGeno is an objective and automated solution for scoring AFLP electropherograms. Our library provides valuable tools for scoring and optimizing AFLP reactions. The user is able to evaluate the quality of the scoring using several statistics that include: the number of bins (with percents of reproducible or filtered bins), the distribution of bin widths, the mean peak intensity, the information content of the dataset, the technical homoplasy rate and the size homoplasy (evaluated with a statistical test, according to Vekemans et al. [13]). These statistics can also be applied while optimizing AFLP reactions or during the screening of AFLP primer-pairs. Several visualisation options (e.g. principal coordinates analyses or “gel-like” pictures) help the user to remove samples that display poor results. In its current version, the package contains exporting functions for a wide panel of genetic analysis programs (the same as in Ehrich [25]). This part of the package is adaptable and will be completed in the further versions of RawGeno.

The library is written in the R CRAN environment and was successfully tested with Windows XP, Vista and Ubuntu 7.10. A graphical user interface allows users to run the package easily but command lines can also be used for coding automation routines.

**Recommendations to optimize the scoring**

The optimization of the scoring of AFLPs requires the manipulation of a large panel of parameters acting during the various stages of the analysis. For the sake of simplicity, we divide the analysis into three steps: the analysis of electropherograms, the definition of bins (including the recording of alleles) and the filtering of non-desirable bins, alleles and samples.

*I. Analysis of the electropherograms*: this preliminary stage consists of detecting and sizing peaks and is achieved using GeneScan V3.1.2 (ABI) or the freely available PeakScanner (ABI). The main problem resides in difficulties in detecting the fluorescence of an amplicon in the electropherogram. Indeed, the algorithms generally use an absolute fluorescence threshold that is applied to the whole sampling. This strategy is necessarily disadvantageous for AFLP profiles that are globally weaker, although the normalization of peak intensities may help to circumvent this problem. As a result, weaker AFLP profiles may significantly accumulate false-negatives, leading to biases in further analyses. Consequently, we propose using mildly permissive settings (e.g. light smoothing and 50 rfu as the fluorescence threshold) during this analysis step along with downstream filtering strategies to increase the dataset quality.

*II. The definition of bins*: this stage is explained in the present study. The main problem lies in choosing a bin width that reflects the best trade-off between oversplitting and technical homoplasy. Our algorithm has the advantage of allowing the user to define both the lower and upper limits of the bin widths. As a consequence, bins can have various widths, giving flexibility to the process. However, if we rely on unpublished results obtained with RawGeno, the results of GeneMapper and the results of Holland et al. [15], we recommend avoiding the use of narrow bins (by defining small “maximum bin width” values) since this setting causes oversplitting. We propose to optimize both parameters with values ranging between 0bp and 2bp for the minimum bin width and 0.5bp and 2bp for the maximum bin width (setting minimum bin width = 0 bp and maximum bin width = 2 bp might be a good starting point according to our preliminary tests). The quality of the scored datasets should then be evaluated by using statistics relying either on replicated samples (e.g. the Bayesian error rates ε1.0 or ε0.1) or the information content per bin, such as the I_bin_ (implemented in the current version of RawGeno). Other quality criteria that measure the robustness of further analyses (e.g. the resolution score of Neighbour-Joining trees or the number of parsimony informative characters [15]) may also be used.

*III. The final filtering of bins*: Since a very large number of bins usually results from the scoring phase (even after having discarded singletons or constant bins), it is necessary to evaluate the quality of each of them by using several filters. For instance, bin reproducibility can be checked if replicated samples are provided. This necessarily requires a large number of replicates to explore a representative range of the genotypes sampled. The unchecked bins (i.e. the bins that were present only in non-replicated samples) can either be conserved or removed from the analysis according to the user settings. Additionally, consecutive bins that show a small size difference can be discarded. (i.e. bins that are located close to each other along the electropherograms decrease the accuracy of bin definitions and peak affiliations). Finally, scoring without regard to the peak intensities may result in the inclusion of false negatives / positives in the final dataset. This situation is especially of concern when AFLP amplifications show ambiguous patterns nearing the intensity detection threshold. Two filters are proposed to deal with this specific problem. Bins that show a low average intensity are considered more likely to contain false negatives and can be discarded [14]. Also, a comparison of peak intensities within each bin helps to avoid the inclusion of false-positives by checking (after normalization of the peak intensities) that peaks within the same bin have a uniform intensity among samples. If requested by the user, the weakest peaks can be discarded from the final scoring. Future versions of RawGeno will provide connection possibilities towards AFLPScore [14], an R CRAN script collection that implements sophisticated filtering solutions (see Appendix). Finally, the application of these filters may depend on the sensitivity of the requested analyses. For instance, diversity measures may require a more conservative estimate of existing peaks, thus more aggressive filtering, while distance based methods seem to be more robust to scoring errors.

**Future plans**

The present version of RawGeno optimizes each step of the analysis independently and this may cause inconsistencies. For instance, non-satisfactory peaks or samples should be removed prior to the bin definition instead of using filtering options only during the third analysis phase. This specific aspect will be investigated in future versions of the library.

Finally, RawGeno represents an opportunity to develop a collaborative and comprehensive tool with experienced users, who are able to edit the library and thereby contribute to its future development.

**Appendix**

Comparison of AFLPScore [14] and RawGeno. Both programs are written in the R CRAN environment and their respective features allow their complementary use. RawGeno includes a scoring solution while AFLPScore proposes sophisticated filtering solutions.

| **Analysis Step** | **RawGeno** | **AFLPScore** |
| --- | --- | --- |
| I. Analysis of the electropherograms | NO (PeakScanner or GeneScan) | NO (GeneMapper) |
| II. Definition of bins | YES | NO (GeneMapper) |
| III. Final filtering of bins | YES (potential connections to AFLPScore in a near future) | YES |
| IV. Quality Check of samples | YES | NO (Achieved prior to the analysis). |
