# Supplementary figures and images for: Evaluating the impact of scoring parameters on the structure of intra-specific genetic variation using RawGeno, an R package for automating AFLP scoring
Source: BMC Bioinformatics. 2009 Jan 26;10:33. doi: 10.1186/1471-2105-10-33 (PMC2656475; doi:10.1186/1471-2105-10-33)

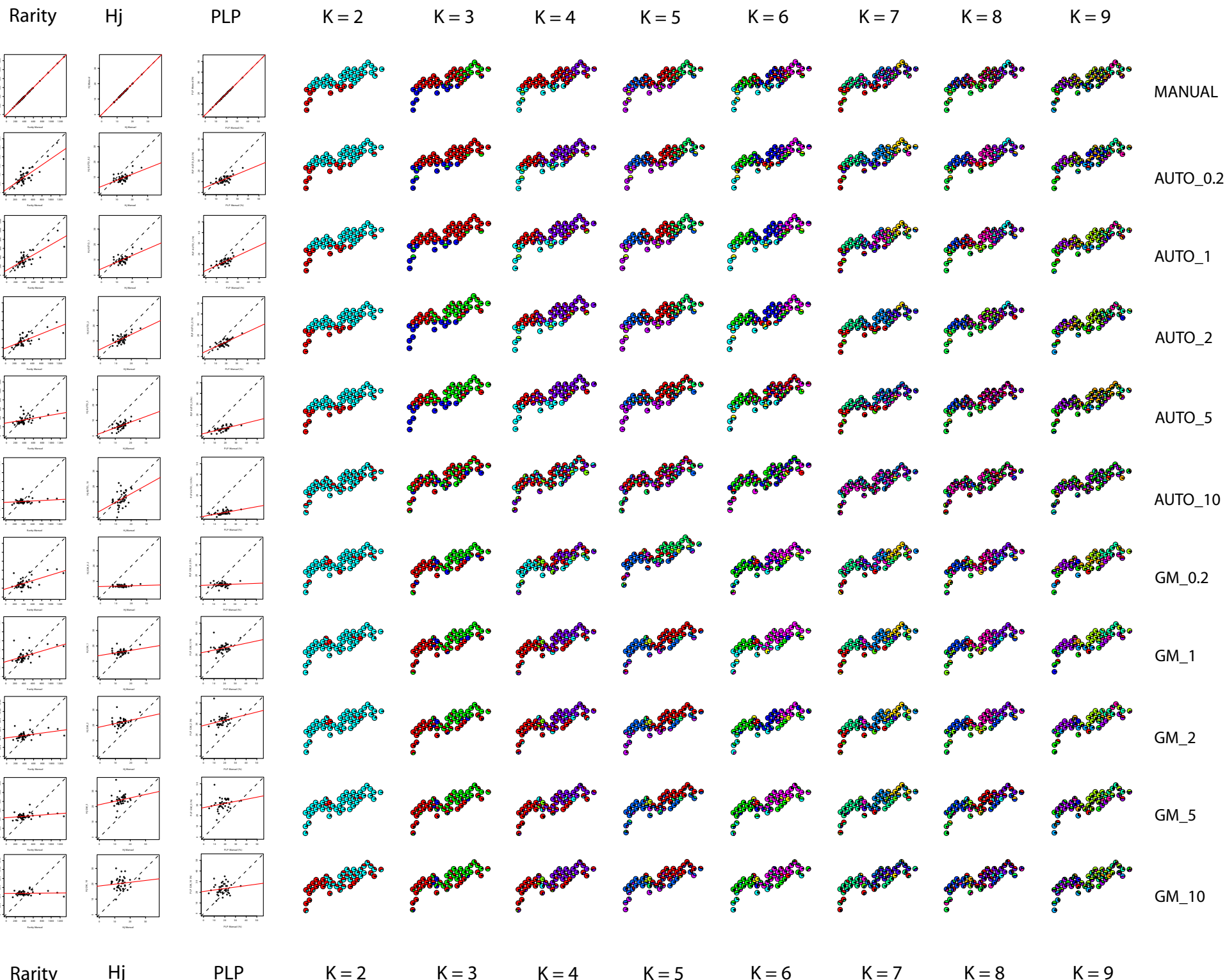

Supplement: Additional file 2 — Spatial genetic structures for all datasets. The graphics are displayed on an array where the lines represent the different datasets (Manual – manual scoring, RG – RawGeno datasets and GM – GeneMapper datasets) and the columns display the different analyses. The three first columns contain the scatterplots of three genetic diversity indices (Rarity – rarity index, Hj – estimated Heterozygosity and PLP – percentage of polymorphic loci). The values obtained by using the automatically scored datasets (displayed on the y-axis) are compared to those obtained with the manually scored dataset (displayed on the x-axis). The red line represents a linear regression between values obtained by both datasets (the Pearson's correlation indices of these regressions are displayed in the Figure 6). The next columns contain individual clustering results (with the number of a priori groups ranging between K = 2 and K = 9). [file 1471-2105-10-33-S2.pdf]

A. PLP

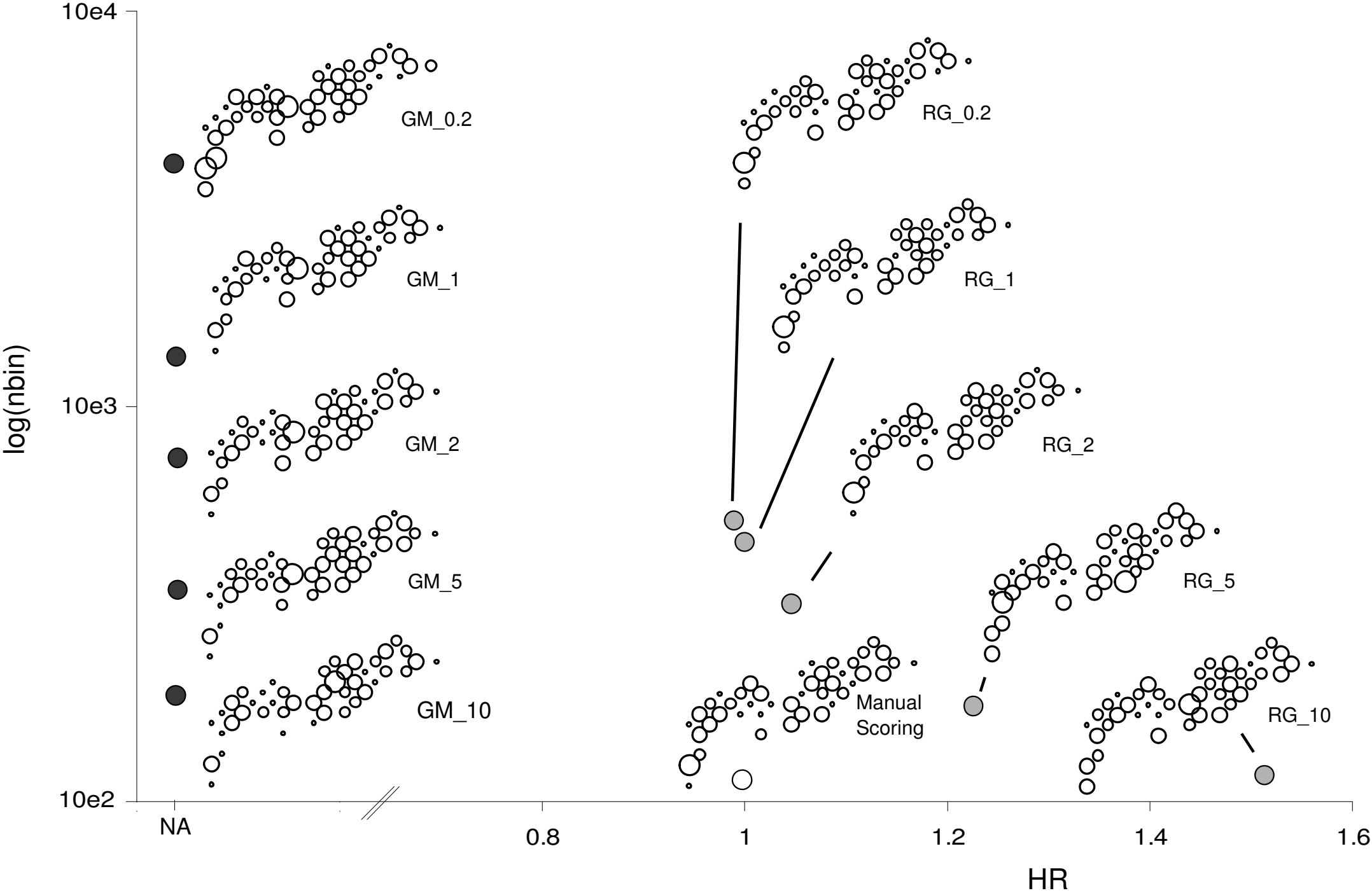

B. Hj

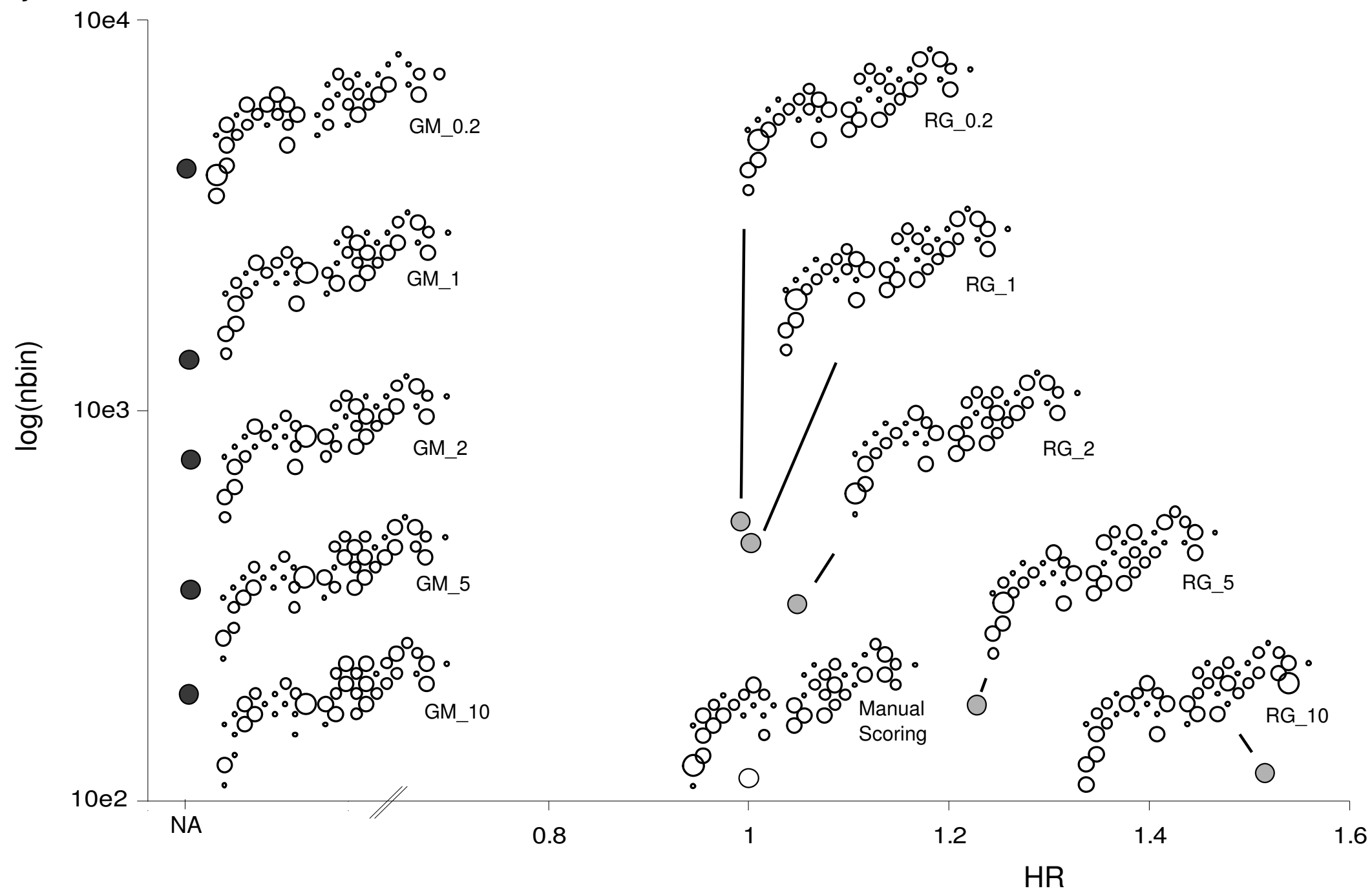

## C. Rarity index

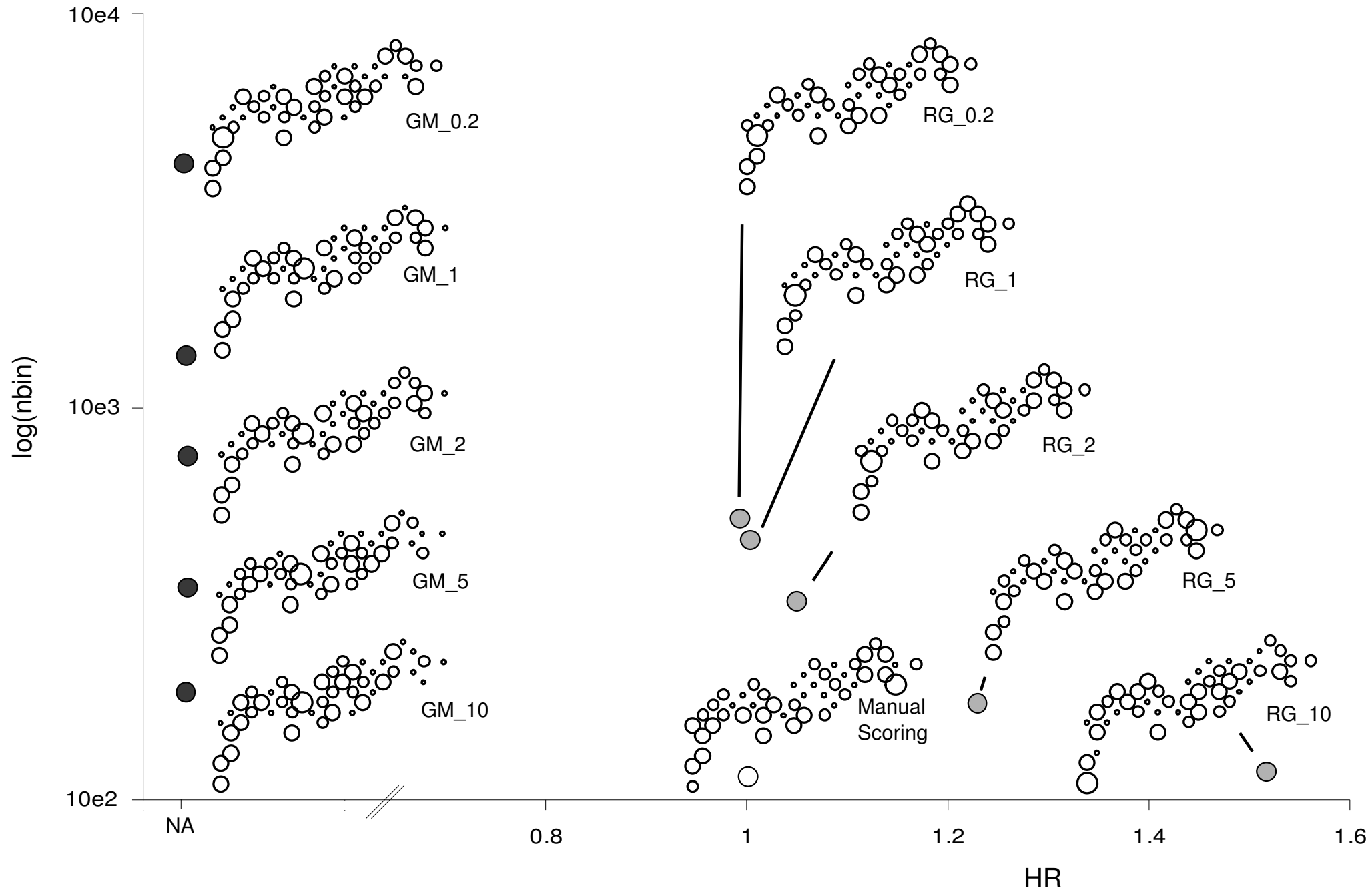

Supplement: Additional file 3 — Effect of the scoring parameters on diversity estimators. Scatterplot of the number of bins (y-axis, log-scaled), according to the mean homoplasy rate (x-axis). The mean homoplasy rate (HR) is defined as the average number of peaks belonging to the same individual that are affiliated within the same bin. The eleven datasets are displayed as dots (in dark grey, GeneMapper datasets labelled with "GM" as prefix; in light grey, RawGeno datasets labelled with "RG" as prefix and in white, manual dataset) and as maps on the scatterplot. The maps represent C. uniflorum populations with circles. The radius of circles is a function of the measured diversity. A. Percentage of polymorphic loci (PLP). B. Estimated Heterozygosity (Hj). C. Rarity index (Rarity). [file 1471-2105-10-33-S3.pdf]
